# “Under Pressure” – How fungi evade, exploit, and modulate cells of the innate immune system

**DOI:** 10.1016/j.smim.2023.101738

**Published:** 2023-03

**Authors:** Theresa Lange, Lydia Kasper, Mark S. Gresnigt, Sascha Brunke, Bernhard Hube

**Affiliations:** a Department of Microbial Pathogenicity Mechanisms, Hans Knoell Institute, Jena, Germany; b Junior Research Group Adaptive Pathogenicity Strategies, Hans Knoell Institute, Jena, Germany; c Institute of Microbiology, Friedrich Schiller University, Jena, Germany

**Keywords:** Human fungal pathogens, Immune evasion, Evolution, Macrophages, Host-pathogen interactions

## Abstract

The human immune system uses an arsenal of effector mechanisms to prevent and counteract infections. Yet, some fungal species are extremely successful as human pathogens, which can be attributed to a wide variety of strategies by which these fungi evade, exploit, and modulate the immune system. These fungal pathogens normally are either harmless commensals or environmental fungi. In this review we discuss how commensalism, but also life in an environmental niche without human contact, can drive the evolution of diverse and specialized immune evasion mechanisms. Correspondingly, we discuss the mechanisms contributing to the ability of these fungi to cause superficial to life-threatening infections.

## Highlights

• Commensal-host co-evolution facilitates development of immune evasion strategies.
• Immune evasion strategies can evolve without a mammalian host in the environment.
• An ability to sense host-derived signals enables adaptive pathogenicity strategies.
• Survival in phagocytes relies on modulation, adaptation, and stress resistance.
• Successful infections of intracellular niches require exit strategies.

## 1 Introduction

Compared to bacteria or viruses, only a small number of fungal species have entered close relationships with mammalian hosts, ranging from symbiotic, commensal to pathogenic (both obligate and facultative) [1], [2], [3]. For those that took such a step, despite the high mammalian body temperature and the immune system, the benefits must have outweighed the costs. One benefit may be the access to plentiful nutrient resources available in a mammalian host (reviewed for bacteria in [4]).

The constant host-fungus interplay drives evolution and adaptation on both sides. While symbiotic interactions are beneficial for both hosts and fungi, commensal relationships are mostly seen as beneficial for the fungi, but not harmful for the host. However, commensal relationships may have trained the hosts to evolve efficient defense mechanisms against fungal pathogens [5], [6], [7]. In line with this, host defense mechanisms that were present early in the evolution of multicellular organisms as well as sophisticated adaptive immune responses capable of generating immunological memory are induced during infection and disease, but also intermittently during commensalism [8].

One ancient mechanism still forms a first line of defense against fungal pathogens: Compartmentalization of the invader, where specialized innate immune cells internalize or phagocytose the fungal pathogen, in order to take the “fight to the inside” [8], [9]. These professional phagocytes can be, for instance, macrophages and neutrophils that both are crucial for clearance of tissue-invading fungi [9]. Recognition of fungal-specific molecules by phagocytes leads to the internalization of the fungal cells [9], [10]. Consequently, the fungi become entrapped in a phagosome that matures into a specialized antimicrobial organelle, the phagolysosome [10]. Depending on the type of immune cell, fungal cells are exposed to a myriad of antimicrobial mechanisms within this organelle, for example, hydrolytic enzymes, reactive oxygen and nitrogen species, metal toxicity, and antimicrobial peptides [10], [11], [12]. In addition to these active antifungal mechanisms, phagocytes also restrict essential nutrients and trace elements in a process which has been termed “nutritional immunity” [13], [14], [15]. Additionally, chemokines and cytokines are released to promote the recruitment and activation of additional innate immune cells and, in the long-term, activate adaptive immune responses [8], [16].

These host defense mechanisms exert a strong evolutionary pressure on fungi to survive within their mammalian host. Therefore, fungi that have co-evolved with their host in commensal or pathogenic relationships developed specialized immune evasion strategies. Yet surprisingly, many fungi from environmental niches without frequent host contact employ similar strategies.

In this review, we will focus on host-associated and environmental human fungal pathogens. We will discuss how immune evasion strategies may have evolved, and how these fungi evade, exploit, and modulate phagocytic cells of the innate immune system. In addition, we introduce the concepts of “cross-adaptation” and “cross-protection” to describe common phenomena in the evolution of fungal pathogenicity in mammals.

## 2 Evolution of immune evasion

The majority of human fungal pathogens live in the environment, including *Cryptococcus*, *Aspergillus,* and *Histoplasma* species [3], [17], [18]. Yet several fungi like certain *Candida* species have most likely been permanently associated with humans as harmless commensals since the evolution of early hominids [19].

Independent of their origin, species like *Candida albicans, Cryptococcus neoformans*, *Histoplasma capsulatum*, and *Aspergillus fumigatus* can cause life-threatening disease and diseases that significantly impact quality of life affecting more than 150 million people each year [20], [21]. Major predisposing conditions for such infections are immunosuppressive medications and diseases such as AIDS, disruption of mucosal barriers due to anti-cancer therapies or surgery, or the use of catheters that enable access to the bloodstream [20], [22]. Imbalance of the microbiota due to long-term treatment with broad-spectrum antibiotics, which favors fungal overgrowth on mucosal surfaces or the skin, also constitutes a risk factor [22], [23].

How do these fungal species cause infections? In the case of environmental fungi, spores or mycelial fragments are often inhaled by humans and enter the host *via* the lungs [20], [24], [25]. In susceptible individuals, these environmental pathogens can cause local pulmonary infections or invade deeper tissue and disseminate trough the body, leading to severe systemic infections [20], [24], [26]. *C. neoformans* and *H. capsulatum* are capable of invading the central nervous system [27], [28], whereas *A. fumigatus* can cause severe damage to the lungs of infected patients [25], [26].

In contrast, *Candida* species like the polymorphic yeast *C. albicans* colonize human mucosal surfaces such as the oral cavity, vagina, and gastrointestinal tract, and are normally kept in check by immune surveillance mechanisms and the microbiota [17], [29], [30], [31]. However, when the host immunity or epithelial barriers are compromised, these opportunistic pathogens can invade tissues, translocate into the bloodstream, disseminate throughout the body, and infect vital organs [29].

Both, environmental and endogenous pathogens constantly need to adapt to their niches to secure their survival. Interestingly, certain adaptations have evolved several times due to similar evolutionary pressures in otherwise different environments [2]. In a striking example of such convergent evolution [32], [33], two completely unrelated pathogens, the environmental fungus *C. neoformans* and the causative agent of bubonic plague, the bacterium *Yersinia pestis*, show similar strategies for immune evasion [32]. Both species are facultative intracellular pathogens and proliferate in phagocytes as part of their pathogenic life style [32]. This strategy has evolved in many more bacterial and fungal species [34], [35], which will be discussed later in this review for pathogenic fungi (see Section 3.2). Especially for environmental fungi that are not constantly associated with the mammalian host, such immune adaptations may be accidental and rather give the pathogen a fitness advantage in their environmental niche [32], [35], [36]. Endogenous fungi like *C. albicans*, however, co-evolved with the selection pressures present in their humans host [3]. It can thus be proposed that these fungi have “learned” adaptations during their commensal lifestyle that can also benefit them during infections [3].

In the following sections we will discuss both adaptation scenarios and how these give rise to immune evasion mechanisms of human pathogenic fungi.

### 2.1 Accidental virulence and immune evasion

**Fig. 1:**
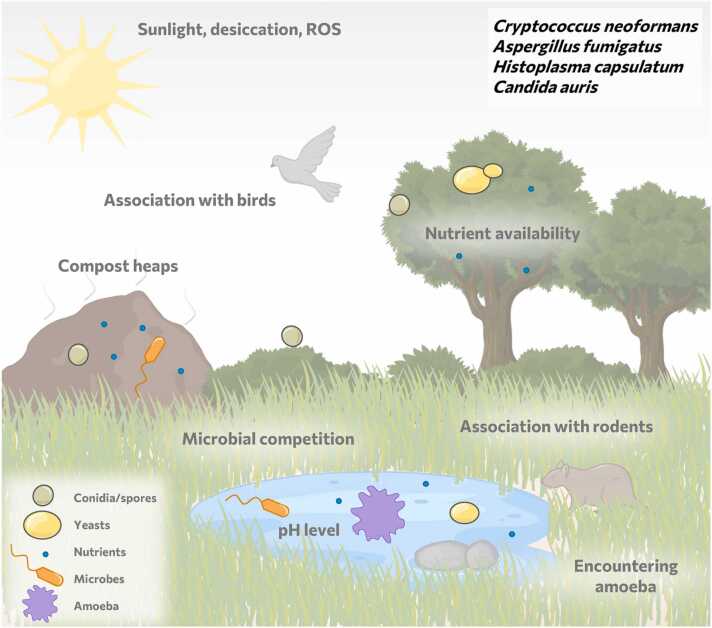
Environmental virulence school. In the environment fungi are exposed to changing temperatures as for instance in compost heaps, pH and osmolarity changes, reactive oxygen species (ROS), sunlight and amoeboid predators. In addition, they have to “learn” how to adhere to surfaces, form biofilms, acquire nutrients, and compete with other microbes. Some environmental fungi also associate with birds or mammals such as rodents.

Environmental fungi likely evolved their virulence and immune evasion strategies by coping with stresses in their environments, like temperature, pH, and osmolarity, that resemble those of the human immune system [3], [36]. They also learned how to adhere to surfaces, form biofilms, compete with bacteria, and acquire essential nutrients [3]. Therefore, the environment functions as a virulence and immune evasion training ground and can be seen as an "environmental virulence school" (Fig. 1) [3], [32], [34]. A notable characteristic that distinguishes human fungal pathogens from other environmental fungi is their capacity to thrive at mammalian body temperature. This thermal barrier is generally thought to protect humans from infections by most environmentally adapted fungal species [37], [38], [39]. However, some fungi, including *A. fumigatus,* evolved mechanisms to grow in natural high-temperature niches such as (self-heating) compost heaps [25], [26], [40]. This can be considered a cross-adaptation to the human host. Similarly, it has been proposed that global warming may have driven adaptations in *Candida auris*, which allow this globally emerging fungal pathogen to survive in humans [37].

Another example of the environmental virulence school can be seen in cell wall structures of environmental fungi which protect against environmental stresses but also against host immune responses. The capsule of *C. neoformans*, for example, protects against desiccation and oxidative stress in the environment [36], [38], [41], [42], [43] but also against exposure to reactive oxygen species produced by immune cells [44]. In addition, *C. neoformans* melanin confers protection against high temperature, sunlight, and oxidative stresses in the environment [36], [45], [46], however, it also renders *C. neoformans* less susceptible to ingestion and killing by macrophages, and protects it from immune-generated oxidative stresses [47], [48]. The melanin layer of *A. fumigatus* similarly shields it against sunlight or radiation and preserves from desiccation [49], [50], [51], conferring a cross-protection against host immune responses [26], [46], [52], [53], [54], [55], [56], [57]. Additionally, *A. fumigatus* possesses a hydrophobic rodlet layer that most likely facilitates air-borne dispersal of conidia and mediates growth of aerial hyphae from moist environments [51], [58], [59]. This layer, however, also shields it from recognition by the immune system [60] (see Section 3.1).

The environmental niche also harbors predators that have driven the development of evasion mechanisms [34], [38]. Such predators can be small animals (such as nematodes) or free-living amoebae [38]. The latter employ feeding mechanisms that are strikingly similar to phagocytosis by mammalian innate immune cells [38], [39], [61], [62]. Environmental fungi evolved strategies to resist ingestion by amoebae or survive intracellular killing, which mediate cross-adaptation to human phagocytes [18], [36], [38], [63], [64]. This could have driven the evolution of the facultative intracellular lifestyle of *C. neoformans*, but also other fungi such as *H. capsulatum* [34], [36], [38]. Similarly *A. fumigatus* was suggested to be “trained” by ameboid predation to resist phagocytosis and killing by macrophages [61].

Collectively, the “dual use” [36] of strategies evolved to cope with environmental abiotic and biotic stresses presumably cross-adapted fungal pathogens to stresses imposed by the human immune system. Furthermore, some of these environmental fungi are also infrequently associated with animal hosts. Examples include *H. capsulatum*, which is intermittently associated with rodents, and *C. neoformans*, which has been isolated from bird droppings [18], [65], [66], [67], [68], [69]. Thus, these fungi may have benefitted from adaptation to environmental as well as host-associated pressures.

### 2.2 Learning immune evasion from the host defense

**Fig. 2:**
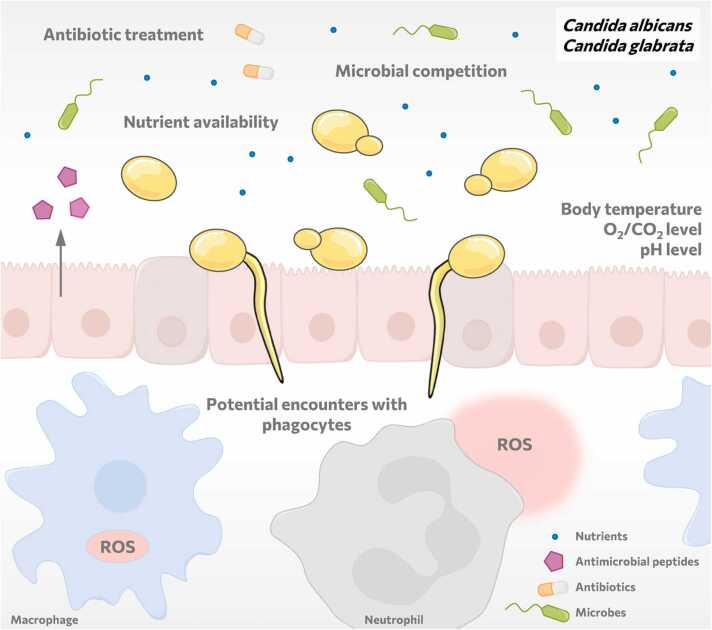
Commensal virulence school in the gastrointestinal tract. In the host fungi have to “learn” how to adhere to host surfaces, to form biofilms, to acquire nutrients and to resist physiological temperatures and other niche-specific stresses. In addition, fungi have to resist competing microbes, antifungal or antibiotic treatments, and intermittently immune cells such as phagocytes.

To co-exist with their host, commensal fungi like several *Candida* species require a certain degree of immune evasion or tolerance [8]. These strategies are believed to be shaped by selection pressures imposed by the host in the so-called “commensal virulence school” (Fig. 2) [3]. They include host surface adhesion, biofilm formation, competition with the microbiome, nutrient acquisition, resistance to physiological temperatures and other physical stresses [3], [70]. In addition, commensals have to resist constant surveillance by the immune system and occasionally face immune cells [3], [29], [70]. These adaptations may also enable commensal fungi to infect the human host when the opportunity arises.

*Candida* species are present in several host niches and thus encounter dynamic and distinct microenvironments during commensalism and pathogenicity [70]. In line with this, these species show species-, stage-, and tissue-specific gene expression patterns, illustrating their ability to rapidly adapt metabolism and physiology to different host conditions [22], [29], [71], [72], [73]. Such rapid adaptations require that *Candida* species “sense” changes and fluctuations in their host niche and respond *via* flexible remodeling and reprogramming of their transcriptional profile (a so-called “adaptive response”) [29], [70]. One example is *C. albicans*’ sensing of low glucose levels, a likely scenario in most host niches, which induces filamentation [70]. Similarly, it can sense the external pH, which activates downstream signaling and affects morphogenesis, biofilm formation, and cell wall remodeling [70]. In addition, sensing of low oxygen levels, as present in the gastrointestinal niche for instance, induces filamentation and promotes masking of immunogenic cell surface compounds [70], [74], [75]. We will discuss in section 3.1 how these modifications influence fungal immune evasion.

Interestingly, counteractivities of the host immunity often happen in a defined sequence, which led to the hypothesis that fungi can predict upcoming immune surveillance stressors [69], [76]. Indeed, signal transduction cascades have been characterized in *C. albicans* that allow the “anticipation” of future challenges based on host molecules and markers [69], [76], [77], [78], [79]. Such anticipatory mechanisms are characterized by fungal stress responses in absence of an immediate stress [69]. An example for this “adaptive prediction” is *C. albicans*’ response to glucose upon entering the bloodstream: The fungus upregulates oxidative and osmotic stress responses, which will be beneficial during upcoming encounters with phagocytes [77], [79], [80]. Notably, anticipatory responses were also found in environmental fungi such as *C. neoformans*. This fungus exploits serum, elevated carbon dioxide concentrations, and body temperature, all present in the human host, to activate its melanin, capsule, and titan cell formation, which provides protection against the host immunity [69], [81], [82], [83]. Moreover, specific transcriptional patterns to cope with increased stress resistance have been characterized in *C.neoformans* grown at 37 °C [84].

It has been proposed that the accumulation of several of these anticipatory protective responses might have led to the emergence of so-called “core stress responses” (CSR) [69]. This concept suggests that when fungi sense a stressor in their environment, they commonly induce a core set of stress genes, providing a cross-protection against a variety of stressors [85]. This also includes protective genes against immune surveillance mechanisms [69]. So far, no CSR has been observed in *C. albicans* [86], [87]. This might be because the evolutionary advantage is insufficient, as inducing a core set of genes to multiple stressors must provide a fitness advantage that outweighs the energetic costs [69], [88].

Although the commensal virulence school is a convincing concept, we cannot exclude the possibility that host-associated commensals such as *Candida* species evolved their immune evasion strategies, at least partially, in the environment prior to encountering the human host for the first time. *C. albicans* has no specific nutrient requirements that would prevent it from existing in the environment [29]. In fact, three recent studies isolated *C. albicans* strains from environmental niches such as soil, trees, and pigeon droppings [68], [89], [90]. Supporting this, its environmental relative *C. auris* might be a recent example of a species that “jumped” from an environmental niche to also colonize and infect humans [91]. *C. auris* has been shown to survive predation by amoeba, and potentially, the evolution of these evasion strategies now facilitates its survival in mammalian hosts [91], [92], [93].

## 3 Host adaptation and immune evasion mechanisms

Fungal immune evasion strategies may partly explain the severity of infections and better knowledge about them might allow targeting these interactions for treatment. The presence of efficient immune evasion strategies, however, does not necessarily mean that a fungus is a successful pathogen. *A. nidulans*, a relative of *A. fumigatus*, is less efficiently recognized and cleared by the immune system [94]. However, this fungus is the cause of only a minority of aspergillosis cases [95]. Similarly, *C. dubliniensis*, the phylogenetically closest species to *C. albicans*, is much less virulent although it is able to colonize host mucosal surfaces like *C. albicans* [17]. Therefore, immune evasion strategies can be observed in both mild and severe fungal pathogens. In this section, we introduce immune evasion strategies of environmental and commensal fungi, and in particular focus on how they avoid, escape, and even exploit host responses and phagocytes.

### 3.1 Catch me if you can – Resistance and avoidance of immune recognition

#### 3.1.1 Avoiding to be attacked by the immune system (Fig. 3)

Immune recognition of microbes relies on the recognition of specific molecular patters unique to the pathogen (Pathogen/microbe-associated molecular patterns, PAMPs/MAMPs). For fungal pathogens, the major PAMPs are polysaccharides of the cell wall, including mannan, β-glucan, chitin, α-glucan, galactomannan, and galactosaminogalactan [9], [96], [97]. However, the extent to which these molecules activate protective immune responses or alternatively manipulative responses is highly variable. Immune cells recognize PAMPs using specialized receptors (pattern recognition receptors, PRRs) [98], [99], [100]. Alternatively, soluble host defense factors, such as antibodies and the complement system, can recognize foreign molecules and play a crucial role in opsonization [8].

The key aspect underlying the functionality of the immune system is the capacity to discriminate self- from non-self-molecules [100]. Parasites exploit this by producing host-mimicking molecules that show molecular or structural homology with their mammalian host (molecular mimicry) [101], [102]. Recognition of these molecules is not beneficial for the host as cross-reactivity with self-molecules has been implicated in detrimental auto-immune response [103]. While molecular mimicry, to our knowledge, has not been observed in fungal pathogens, they use a similar strategy by binding host (self-) molecules and thereby hide or mask their immunogenic epitopes*. C. albicans*, for example, can bind factor H, which inhibits complement activation [104] and was predicted to influence self vs. non-self-discrimination by the host [105]. Such strategies are, however, unlikely in environmental microbes that did not have the chance to co-evolve with the host. Surprisingly, *A. fumigatus* also has the capacity to bind complement inhibitors [106].

Nevertheless, environmental fungi excel in masking immunogenic epitopes by producing non-immunogenic molecules that form a physical barrier on top of the epitopes. One example for this strategy is provided by the spores (conidia) of *Aspergillus* species that are covered by hydrophobic rodlets [60] and DHN-melanin [47], [48]. *A. fumigatus*’ rodlet layer renders fungal conidia immunogenically silent by masking immunogenic cell wall components [60]. The melanin layer of *A. fumigatus* protects it against reactive oxygen species released by immune cells, modulates the immune response, prevents immune recognition, and even manipulates phagocytosis by human monocytes and macrophages [46], [52], [53], [54], [55], [56], [60]. Interestingly, recently a receptor has been discovered that actually mediates recognition of DHN-melanin and is crucial for an efficient defense against *A*. *fumigatus* [107].

Comparable to *Aspergillus* species, other environmental fungal pathogens that are transiently host-associated also display intricate strategies to mask their immunogenic epitopes. This includes the polysaccharide capsule of *C. neoformans*, which is a major virulence attribute [41]. The capsule comprises mostly the poorly immunogenic glucuronoxylomannan polysaccharides that not only shield underlying PAMPs from recognition, but also modulate inflammatory responses [41]. Similarly, *H. capsulatum* masks its highly immunogenic β-glucan by the less immunogenic cell wall polysaccharide α-glucan [108]. Evading immune recognition is also mediated by dynamic changes in the cell wall architecture. For example, *C. albicans* has an extremely dynamic cell wall architecture that changes depending on its environment [109], e.g., in response to lactate [110]. While all major components of the *C. albicans* cell wall (β-glucan, chitin, and mannan) can be recognized by the immune system [111], the different cell types of the immune system that are engaging the fungus also impact whether a cell wall molecule acts as a PAMP or as a masking molecule [112]. For example, mannan is often considered to be masking the immunogenic PAMP β-glucan, however, the efficiency of *C. albicans* phagocytosis depends on the type of mannan present [113].

The recently emerged *Candida* species *C. auris*, has been observed to interact with the innate immune system distinctly from *C. albicans,* eliciting significantly stronger proinflammatory responses [114]. This was suggested to relate to the unique structural features of mannans in the *C. auris* cell wall [114], which, due to *C. auris’* presumed environmental origin, may not have co-evolved with the human immune system to avoid immune recognition. Interestingly, neutrophil evasion of *C. auris* relies on cell wall mannosylation mediated by *PMR1* and *VAN1,* while these genes do not impact interaction of the commensal species *C. albicans* and *C. glabrata* with neutrophils [115]. As discussed in 2.1 and 2.2, this divergence may be due to training of immune evasion in the environmental (*C. auris*) or in the commensal virulence school (*C. albicans* and *C. glabrata*).

Supporting the importance of a dynamic cell wall, *C. albicans* can remove the immunogenic β-glucan depending on the host environment using an exoglucanase, a process which has been termed “β-glucan shaving”, promoting immune evasion [116]. Similarly, *H. capsulatum* uses a β-glucanase to reduce its β-glucan exposure [117]. A resembling mechanism is employed by *C. neoformans*. This fungus uses a chitosan deacetylase to alter its cell wall exposure, making itself less susceptible to immunosurveillance mechanisms [118]. In addition, coordinated morphological changes of fungi, such as the yeast-to-hypha transition of *C. albicans*, also influence the cell wall composition and, hence, immune evasion [119]. Thus, by maintaining a dynamic variability in PAMP exposure through a plethora of different mechanisms, immune recognition can be manipulated and avoided.

Finally, immune evasion may be facilitated by degradation of immune molecules. For example, secreted aspartic proteinases (Saps) of *C. albicans* have the potential to hydrolyze diverse immune proteins such as antibodies and complement factors (reviewed in [120]). *A. fumigatus* can also degrade host factors as well as the host pattern recognition receptors dectin-1 and dectin-2 mediating its recognition [121], [122].

#### 3.1.2 Take someone of your own size

**Fig. 3:**
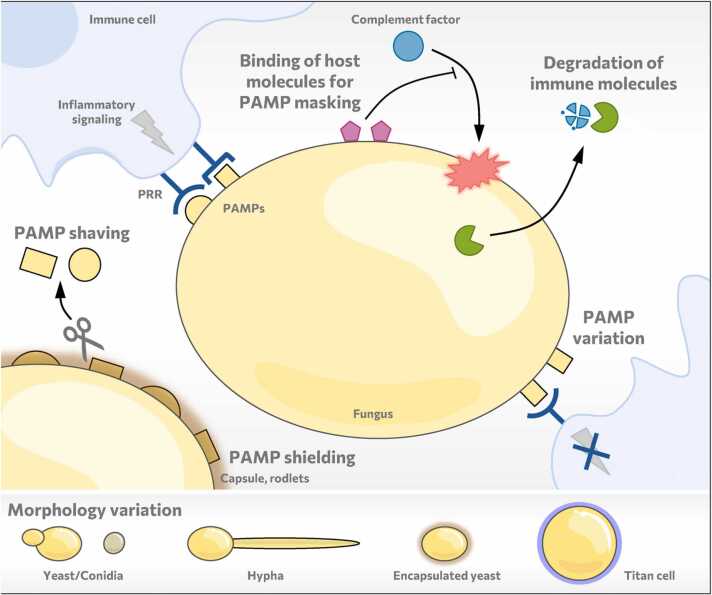
Avoiding immune recognition. Human pathogenic fungi exhibit a wide variety of strategies to avoid immune recognition by pattern recognition receptors (PRR) and opsonization by complement and antibodies. Opsonization can be avoided through binding host molecules that inactivate complement or by releasing proteases that can degrade host proteins. Avoiding the recognition of pathogen-associated molecular patterns (PAMPs) is achieved by masking or shielding using non-immunogenic epitopes. Besides, PAMP variability and the expression of PAMPs that induce anti-inflammatory responses reduces the efficiency of immune recognition and activation of inflammatory responses. Similarly, shaving of immunogenic PAMPs facilitates immune evasion. Additionally, (lower panel) fungal growth in different morphologies affects immune recognition and phagocytosis. Hypha formation as well as formation of titan cells, for example, impairs macrophage phagocytosis. Moreover, covering immunogenic molecules as in the case of conidia or encapsulated yeasts, renders fungi immunogenically silent.

While single bacteria can be easily engulfed by phagocytes of the innate immune system, the growth of fungi in different morphologies complicates antifungal host defense (Fig. 3). In fact, shape drastically affects phagocytosis efficiency [123], [124], [125], whereas a large particle size simply leads to an inability to engulf the object [124]. In line with this, the spatial orientations of *C. albicans* hyphae are also important determinants of their internalization rate by macrophages [126].

Yet when hyphae are too long to be completely engulfed, sealed tubular phagosomes, named "frustrated phagosomes", are generated [12], [127]. This presumably happens to generate a partially contained environment where antimicrobial effector molecules can accumulate. Nevertheless, macrophages have also been observed to fold (partially) internalized hyphae depending on actin-myosin cytoskeleton dynamics, which can facilitate the complete engulfment of hyphae [128]. While filamentous growth may play a role during colonization of the oral cavity by *C. albicans* [129], the increased difficulty for the immune system to clear hyphae compared to yeast may have been an additional evolutionary pressure towards maintaining filamentation for this fungus. This is supported by the notion that genes required to counteract immunological effector mechanisms, such as the gene encoding superoxide dismutase 5 (*SOD5*) (see Section 3.2), are specifically expressed during hypha formation [130].

Another morphological solution to overcome phagocytosis is the formation of titan cells by *C. neoformans*. Titan cells are difficult to phagocytose due to their sheer size and additionally show increased resistance to oxidative stress [131]. These cells have been postulated to play a crucial role to establish colonization of the mammalian lung. Contrary to this *C. neoformans* can form a small cell variant called “seed” cells that is critical for extrapulmonary organ entry and facilitates dissemination [132], [133]. Additionally, this morphotype mediates tissue-resident macrophage phagocytosis, which enhances fungal burden in the respective organs potentially by facilitating the organ entry [133].

Finally, biofilm formation is probably one of the most often convergently evolved strategies in microbes that allows specialization and increased resistance to stresses [134]. Naturally, the formation of huge microbial communities in biofilms complicates clearance by phagocytes of the innate immune system [135].

### 3.2 I want it all – Manipulation and exploitation of immune cells

**Fig. 4:**
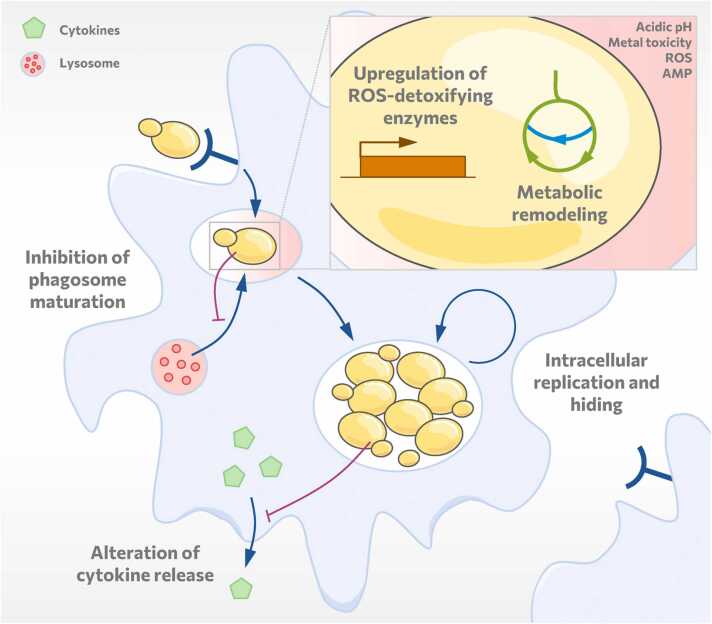
Manipulation and exploitation of phagocytes. After phagocytosis fungal pathogens reside in the maturating phagosome. In order to adapt to the limited nutrients intracellularly, fungi up-regulate alternative carbon source metabolism pathways. In addition, they inhibit phagosome maturation, up-regulate reactive oxygen species (ROS)-detoxifying enzymes, and alter the cytokine release of immune cells. By counteracting these antifungal measures, fungi can create an intracellular compartment in which they can replicate and hide, shielding themselves from other immune cells.

Even after phagocytosis, fungi can alter, modulate, and resist the immune cell's activities and responses (Fig. 4). A common – and often important – first fungal-driven event after phagocytosis is the inhibition of phagosomal acidification and phago-lysosomal fusion. Among the major human fungal pathogens, *C. neoformans* [136], *H. capsulatum* [137], [138], *C. glabrata* [139], *C. albicans* [140], and *A. fumigatus* all modify the intraphagosomal pH to different extents. Interestingly, all these fungi seem to employ different properties to achieve this goal, using for example specific cell wall polysaccharides and urease activity (*C. neoformans*) [141], [142], leucine catabolism (*H. capsulatum*) [143], mannosylated proteins and protein trafficking (*C. glabrata*) [144], [145], carboxylic and amino acid metabolism (*C. albicans*) [140], [146], [147], [148], [149] or melanin (*A. fumigatus*) [150]. The host-side events leading to pH manipulation similarly differ, and e.g., *C. neoformans* initially allows phagolysosomal fusion, but later increases the pH by damaging the phagolysosomal membrane [151], [152] to allow influx of cytoplasm and its nutrients [153]. Similarly, *C. albicans* hyphal extension facilitates phagosomal rupture and allows transient access to the cytoplasm and a rise in pH. Here, constant lysosomal fusions are required to repair the phagolysosomal lesions quickly [154], [155], [156], [157].

The constant barrage of reactive oxygen species is countered by an often-dedicated set of detoxifying enzymes and molecules. Of special note is the superoxide dismutase, Sod5, of *C. albicans*. This enzyme is the first one to be described that requires only copper, not copper and zinc like most superoxide dismutases [158]. This seems to be a striking example for an adaptation to the macrophage phagolysome, where copper is often actively transported into the fungus-containing phagosome and zinc is removed [159]. Indeed, other fungi like *C. auris* or *C. tropicalis* contain similar Cu-only superoxide dismutases [160]. Similarly, the sole catalase of *C. glabrata*, Cta1, is highly effective and protects the fungus from even higher oxidative stress levels than *C. albicans* can tolerate [161]. Finally, the melanins of many fungi are effective scavengers of reactive oxygen species [162], [163].

Another stress within the phagosome, the limited nutrient availability, is countered by different fungal strategies. An important contribution comes from the glyoxylate shunt. This bypass of the tricarboxylic acid cycle enables fungi to use two-carbon compounds like acetate when glucose is limited. Such compounds can be derived e.g., from fatty acids and enable the fungi to use their lipid resources for survival inside the phagosome. In fact, the glyoxylate shunt has been shown to be important for virulence in many fungal species [164], [165], [166], [167]. Other storage molecules may play similar roles during macrophage interactions, such as trehalose in *C. glabrata* [168].

Using a different strategy, *C. glabrata* can acquire one of the important micronutrients, iron, in macrophages that are activated by type I interferons. In these cells, iron is not sequestered from the *C. glabrata*-containing phagolysosome, allowing growth and ultimately persistence in the host [169]. Importantly, type I IFN responses are elicited in response to *C. glabrata* and *C. albicans* [170], [171] and *Candida* species infection of epithelia triggers type I interferon signaling [172].

While these modifications of the phagosome are short-term solutions for many fungi to alleviate stress, some species can make the modified phagosome their home. A classic example is *C. neoformans*, which actually grows better at the still-lower pH of its modified phagosome than in some culture media [173]. This fungus can survive intracellularly within macrophages for long periods of time, and it has been suggested that it even uses them as "Trojan horses" to cross the blood-brain barrier [174], [175], [176], [177]. By hiding within macrophages, it would also be protected from other aspects of mammalian immunity that can actually harm the fungus such as neutrophils [178]. Similar strategies seem to be employed by other fungi: *H. capsulatum* delays death of neutrophils and mononuclear cells, by inhibiting apoptosis [179]. On the contrary, during early pulmonary infection *H. capsulatum* can actively induce apoptosis in macrophages, which subsequently triggers IL-10 release, preventing apoptosis of infected neighboring phagocytes [180]. These macrophages serve as a protected environment, which allow *H. capsulatum*’s dissemination from the lung to other organs [181].

In recent years, it has been suggested that a similar strategy could also be employed by *C. glabrata* [182]. *C. glabrata* differs in its infection strategies from its relative *C. albicans* as it cannot form hyphae [183], [184]. In contrast to *C. albicans*, which escapes from macrophages within hours (see Section 3.3), *C. glabrata* can persist within macrophages for days [139]. The potential role of this interaction during infection is still under investigation.

In addition to ensuring intracellular survival by directly modifying their host immune cell environment, fungi can also manipulate cytokine secretion of host cells to shape the next steps of the immune reaction. There are many examples for specific or broader pathogen-driven alterations in the cytokine profiles of phagocytes after ingestion of fungi. Frequently, these seem to involve a repression of pro-inflammatory cytokines that would attract more immune effector cells like neutrophils. For example, macrophages infected with *C. glabrata* produce very little pro-inflammatory cytokines such as TNF or IL-6 and the neutrophil chemoattractant IL-8 compared to e.g., *S. cerevisiae* or *C. albicans* [139]. Interestingly, in contrast to other cytokines, GM-CSF is produced at high levels by *C. glabrata*-infected mice [185], and specifically macrophages [139] and epithelial cells [186], [187]. One function of GM-CSF is the attraction of monocytes and activation of macrophages [188]. It is tempting to speculate that this may benefit the macrophage-resistant *C. glabrata* by attracting suitable host cells. A recent publication in fact revealed that a large proportion of macrophages exhibits enhanced kinesis towards *C. glabrata* (and *C. albicans*) in comparison with other pathogenic fungi [189]. Moreover, *C. glabrata* is found surrounded by monocytes in experimental murine infections [185].

For *C. neoformans*' modulation of the immune response, the previously mentioned capsule again plays an important role. Extracellularly, it is recognized by TLR2 and 4 and induces the nuclear translocation of NFκB, but it masks the signal required for MAPK signaling [190]. This lack of signaling prevents the secretion of TNF, which is required for nitric oxide synthase (NOS) induction [191] and ultimately clearance of the pathogen. In the long term, intraphagosomal, encapsulated *C. neoformans* increases NFκB presence in the nucleus [192]. This seems counterintuitive, but NFκB forms complexes with the inhibitor IκB, which would normally be exported to the cytoplasm. Intracellular *C. neoformans*, however, somehow subverts this translocation, and the complexes instead fail to induce the NFκB target genes, including NOS [192]. This shows that the same pathway can be targeted by different mechanisms, depending on the stage of infection and of the fungus-macrophage interactions.

NOS is also the target of a *C. albicans* chitin-mediated strategy. In macrophages, NOS competes with arginase for the same substrate, L-arginine, and these enzymatic activities are classical markers of M1 or M2 polarization, respectively [193]. *C. albicans* chitin has been shown to trigger an increased arginase activity, thus leaving NOS without substrate and lowering the macrophages' ability to kill the fungus [193].

Another example are the secreted lipases of *Candida parapsilosis*. Mutants lacking both genes encoding secreted lipases in *C. parapsilosis* induce more pro-inflammatory cytokines in primary macrophages, indicating that these lipases may be involved in suppressing a strong immune reaction after phagocytosis [194]. As many immune modulators are lipid-derived [195], lipase activities may have many more, thus far unknown, immune-modulatory functions in fungi. Similarly, surface-associated proteases of *C. glabrata* suppress production of IL-1β and other cytokines by macrophages, possibly by an indirect mechanism *via* its cell wall composition [196]. Whether similar functions can be found for other extracellular hydrolases, which are abundant among fungi, remains to be seen.

Overall, this non-exhaustive list of examples shows that pathogenic fungi have evolved to not only be passive 'victims' of the mammalian immune system, but instead shape and modulate many aspects of it to their needs. Seen from the pathogen perspective, this makes sense, as immune cells are at least transient parts of their natural environment, to which they adapted over time [17].

### 3.3 Prison break – Escape from immune cells

**Fig. 5:**
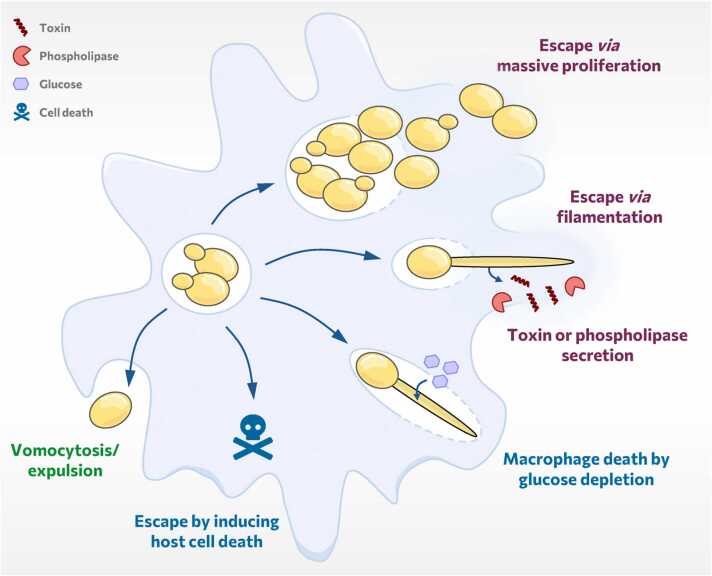
Schematic representation of escape strategies of fungal pathogens from macrophages. These include (i) the active lytic destruction of the host cell by physical forces upon massive proliferation or filamentation as well as toxin or phospholipase secretion (purple), (ii) host cell death induced in response to fungal containment or indirectly due to glucose depletion (blue) and (iii) non-lytic expulsion (vomocytosis) (green).

The ability to survive inside phagocytic cells can be seen as an immune evasion mechanism that allows fungal replication hidden from other immune cells. At a certain point of infection, however, intracellular fungal pathogens have to exit their compartment again. In general, three exit pathways have been postulated for pathogenic microbes: active lytic destruction of the host cell, initiation of a programmed host cell death, and membrane-dependent exit without host cell lysis [197]. Fungal pathogens use all three routes of exit, and most comprehensive knowledge about fungal survival and escape strategies comes from studies on their escape originate from macrophage infection models [9], [181], [182], [198], [199], [200] (Fig. 5).

#### 3.3.1 Causing lytic host cell damage

Lytic escape mechanisms are used especially by fungal pathogens that filament inside macrophages, but some pathogens replicating in the yeast morphology are also able to actively destruct their host cells. For *C. albicans*, rapid escape *via* active host cell damage seems to be a major pathway of macrophage escape. This process largely depends on hypha formation [154]. Elongating filaments generate physical forces which lead to piercing of macrophage membranes and escape within a few hours [113], [155], [201]. On the other hand, *C. albicans* hypha formation is associated with the production of virulence-associated proteins like Sod5 (see 3.1, 3.2). One further hypha-associated factor is the cytolytic toxin candidalysin - one of eight peptides encoded by the strictly hypha-associated polypeptide Ece1. Deletion of the candidalysin-encoding region abolishes the ability of *C. albicans* to damage epithelial cells and significantly attenuates virulence [202], making it the first peptide toxin to be described in a human pathogenic fungus. Candidalysin production by growing macrophage-internalized hyphae causes macrophage cell death, likely *via* direct host membrane damage [203]. While toxin-mediated damage by candidalysin is critical for fungal pathogenicity, candidalysin also activates the immune system. In addition to macrophage damage, candidalysin induces NLRP3 inflammasome activation [203], [204], thus attracting immune cells to the site of infection upon its exit from phagocytes [205], [206].

Similar to *C. albicans*, *A. fumigatus* escapes phagocytes *via* filamentation. *A. fumigatus* conidia internalized by alveolar macrophages can germinate and escape through hyphal growth, eventually leading to lysis of the phagocytic cells [198], [207], [208]. *A. fumigatus* secretes secondary metabolites, including several non-peptide toxins [209], but so far there is no direct connection between toxin production and lytic escape shown for *A. fumigatus*.

Other pathogenic fungi like *C. glabrata*, *C. neoformans* and *H. capsulatum* replicate in the yeast morphology [198], [200], [210], [211]. Yeast proliferation likely causes less rapid physical stress to the host cells, and escape may even be actively delayed enabling the use of phagocytes for intracellular concealment (see Section 3.2). Still, host mechanisms for maintaining phagosome membrane integrity come into play, as shown for phagosome expansion of *C. glabrata*-containing phagosomes [155]. Also, *C. neoformans* cells induce phagosomal membrane permeabilization and the phagosomal damage was associated with host cell apoptosis. Cryptococcal capsular enlargement and phospholipase B1 (Plb1) seem to be involved in these processes [152], [212]. *C. neoformans* can escape from macrophages by inducing lysis, however, the mechanisms behind its lytic escape are not fully elucidated. *C. neoformans* does not produce pore-forming toxins, but Plb1 may contribute to host membrane damage. It may also be possible that replicating cryptococci cause physical stress to phagosome membranes that ultimately leads to rupture [200].

For *H. capsulatum*, a transcriptomic study has highlighted fungal genes that may be important for macrophage survival and replication. Among them was the *LDF1* gene, which encodes a protein required to lyse macrophages, although the underlying molecular mechanism is poorly understood [210].

*C. glabrata* yeast cells survive and replicate within macrophages for days without eliciting strong pro-inflammatory responses, apoptotic or pyroptotic cell death, or substantial host cell damage, suggesting that *C. glabrata* relies on phagosomal persistence rather than immediate phagosomal escape (see Section 3.2) [139], [182], [211], [213], [214], [215]. Escape from macrophages was mainly observed after 2–3 days, when macrophages that contained a high number of replicating cells were bursting [139]. Thus, *C. glabrata*'s mode of macrophage exit is clearly different and delayed compared to *C. albicans*, but the escape mechanisms of this fungus remain unresolved.

#### 3.3.2 Activating host cell death

A second pathway of phagocyte exit employed by many fungal pathogens is the induction of programmed cell death pathways like pyroptosis, apoptosis, and necroptosis [216], [217]. Recently, it has been established that there is extensive cross-talk between these pathways, and the term PANoptosis has been defined, combining key features of pyroptosis, apoptosis, and necroptosis [218]. Both, *C. albicans* and *A. fumigatus* induce PANoptosis in murine bone marrow-derived macrophages (BMDMs), and induction is driven by the innate immune sensor Z-DNA binding protein (ZBP1) [219].

The induction of pyroptosis as a pathway of cell death-induced escape from phagocytes is so far best described for *C. albicans*. *C. albicans*-infected macrophages activate this inflammatory cell death pathway in the first hours of the fungus-macrophage interaction [213], [220]. Pyroptosis induction by *C. albicans* depends on NLRP3 inflammasome activation [213]. The mechanisms of NLRP3 inflammasome activation by *C. albicans* and its importance for anti-fungal host defense has been described in several studies [221], [222], [223]. The connection to host cell death and fungal escape, however, have only been made in the past years. Inflammasome activation is followed by activation of caspase-1. Active caspase-1 cleaves pro-IL-1β and pro-IL-18 cytokines, but also activates gasdermin D (GSDMD), which then forms pores in the plasma membrane enabling the release of bioactive IL-1β and IL-18 and causing cell swelling and lysis. In addition, caspase-4 and − 11 can promote pyroptosis [217]. GSDMD was shown to facilitate *C. albicans* escape from macrophages in a manner that supported both, candidalysin-dependent and -independent escape [224]. Pyroptosis induction by *C. albicans* is a multi-factorial process. Hyphae seem to be an important trigger of NLRP3 inflammasome activation and pyroptosis, as most filamentation-deficient strains are reduced in their ability to induce these processes [213], [220], [225], [226]. Thus, factors additional to hyphal morphology contribute to efficient host cell damage and escape. Current studies suggest that in addition to filamentation, pyroptosis is driven by changes in the fungal cell surface, which lead to the exposure of cell surface components like ergosterol, β-1,3-glucan or N-glycosylated mannoproteins [213], [220], [225], [226], [227]. Exposed cell surface molecules likely also trigger pyroptosis in other fungi, as an acapsular *C. neoformans* mutant was shown to induce the NLRP3 inflammasome and macrophage damage [227], [228].

Phagocyte apoptosis is modified by several fungal pathogens, and in many cases fungal cell surface components are involved as well. Studies with murine macrophage cell lines showed that *C. albicans* cell wall phospholipomannan induces apoptosis [229]. In contrast, two other studies showed anti-apoptotic signaling and inhibition of apoptosis by *C. albicans* mannoproteins in macrophages [230], [231]. Further work will be required to unravel how the ability of *C. albicans* to both induce and inhibit apoptosis impacts on fungal escape from phagocytes. In resting conidia of *A. fumigatus*, DHN-melanin is involved in inhibiting apoptosis pathways [55]. Once conidia germinate, they produce gliotoxin, which inhibits phagocytosis and induces macrophage apoptosis [232], [233]. *C. neoformans* was found to induce apoptotic and necrotic features in infected macrophages, but the type of death pathway induced depends on the macrophage cell type [234]. Other studies showed that fungal capsular polysaccharides galactoxylomannan and glucuronoxylomannan as well as host NF-κB signaling are involved in apoptosis induction [235], [236]. *H. capsulatum*, can induce apoptosis in murine macrophages during early pulmonary infection [180], but also inhibits apoptosis of neutrophils and monocytes in mice [179].

Compared to the other programmed cell death pathways, the role of necroptosis in fungus-phagocyte interaction is less well described. *C. albicans* and fungal PAMPs can directly induce necroptosis in different types of macrophages and dendritic cells through PRR (dectin-1) signaling, and necroptosis has an impact on protection against *C. albicans* infection *in vivo* [237]. In addition, pathology in an *A. fumigatus* extract-induced murine asthma model was reduced in presence of a necroptosis inhibitor [238]. From that study it was, however, unclear which host cells were undergoing necroptosis. Finally, necroptosis was connected to lateral cell transfer of *A. fumigatus* cells. Germinating conidia were transferred from donor to recipient macrophages – a process which was associated with necroptosis and lead to a better control of germination [239].

#### 3.3.3 Non-lytic escape

Internalized fungal cells can be released from macrophages, in a way that the expelling phagocyte and the expelled yeast stay in a morphologically normal and viable form. It is likely that non-lytic escape offers an advantage to the ingested pathogens due to minimizing proinflammatory signaling and thus ensuring that immune activity is kept to a minimal level [198]. This form of non-lytic escape is called extrusion, expulsion, or vomocytosis and is well-described for cryptococcal species [240], [241]. Expulsion of living *C. albicans* [242], *A. fumigatus,* and *A. nidulans* [94] from macrophages has also been described, however, only a minority of ingested cells take this route of escape. Vomocytosis of *C. neoformans* is not only observed in cultured macrophages but also happens *in vivo* in a murine infection model [243]. The mechanisms behind vomocytosis are complex and are reviewed in detail elsewhere [244]. On the fungal side, cryptococcal phospholipase B1 and urease seem to be involved. On the host side, phagosomal pH, actin polymerization, autophagy, the type of macrophage differentiation, and the mitogen-activated kinase ERK5 have an impact [244]. In addition to exiting the phagocyte, expelled fungal cells can also be laterally transferred from infected donor cells to uninfected neighboring cells, a process called dragotcytosis, which may allow continuous concealment from the immune system [245], [246], [247], [248]. Low phagosomal pH and/or high oxidative stress promote dragotcytosis, suggesting that *C. neoformans* triggers this non-lytic exocytosis mechanism to escape from non-hostile phagosome environments [249].

#### 3.3.4 Impact of metabolism on escape

The *C. albicans*-macrophage interaction is an excellent example of how cross-talk between fungal and host metabolism influence immune cell death and subsequent fungal escape. This topic is also reviewed elsewhere [250], [251]. On the one hand, macrophages, particularly when activated, depend on glucose to mount inflammatory response and for their antimicrobial potential [252], [253], but there is a fierce competition for nutrients between infecting *C. albicans* and host cells [254]. Consequently, late macrophage damage by *C. albicans* is not only driven by lysis through virulence factors, but also by macrophage death due to glucose depletion [255]. In addition, macrophage glucose starvation upon *C. albicans* infection activates inflammatory responses [256].

On the other hand, metabolic processes in the macrophage phagosome seem to be essential for the induction and elongation of *C. albicans* hyphae – an important prerequisite for rapid escape. Transcriptomic studies suggest that *C. albicans* encounters a glucose-poor environment in the phagosome and relies on catabolism of alternative carbon sources like amino acids, fatty acids, N-acetylglucosamine, or carboxylic acids to sustain its survival [165].

Recent studies suggest that alternative carbon sources in the phagosome are not only nutrient sources but also priming signals for *C. albicans* [257], [258]. Arginine and proline catabolism are important for filamentation inside macrophage phagosomes [201], [257], and utilization pathways of further alternative carbon sources combine to induce robust filamentation inside macrophages [258].

## 4 Novel findings, outlook and conclusions

As reflected by this review, fungi possess a multitude of strategies to evade and escape the immune system as well as exploit host cells of the innate immune system. Nevertheless, novel studies may reveal that fungi might have even more mechanisms than the ones that have been discovered already.

A recent example provides the discovery of a secreted effector protein in *C. albicans* [259]. Such secreted effector proteins are a common feature of plant fungal pathogens that get recognized by their hosts and initiate pathogen-protective host defense mechanisms, thus facilitating survival of the pathogen [260], [261], [262]. Similarly, a secreted effector protein by *C. albicans* triggers TLR2/4-dependent inflammatory responses [259]. While TLR2- and TLR4-signaling is crucial for an efficient host defense against *C. albicans* [263], inappropriate activation by a secreted effector may dysregulate inflammatory responses and benefit fungal immune evasion (e.g., *via* inappropriate induction of regulatory T-cells) [264]. Analogously, *C. neoformans* secretes an effector protein which was found to reprogram innate immunity *via* TLR2- and TLR4-signaling [265]. In addition, a recent study observed that *C. neoformans* secretes, besides the effector proteins, aromatic metabolites to manipulate the inflammasome activation in macrophages [266]. Moreover, *A. fumigatus*-secreted cell wall polysaccharides can manipulate inflammatory response in favor of the fungus [267], [268]. Collectively, these studies show that pathogen-derived immunomodulators such as secreted effector proteins or molecules provide a new mechanism to evade and exploit the host immune system. However, so far only few studies discovered secreted effectors of human pathogenic fungi and, thus further research is required to identify additional immunomodulatory effectors, which likely exist.

We previously described the concept of sensing host molecules as an approach for fungi to adapt to future stresses inflicted by the host immunity (adaptive prediction, see 2.1). Although the concept is not novel, there are new findings suggesting novel immune evasion mechanisms [79]. A recent study, for instance, revealed that *C. albicans* senses the content of extracellular vesicles, which are released by immune cells, promoting filamentous growth [269]. Similarly, *C. albicans* has been shown to filament in response to the macrophage-derived protein PTMA [270]. Filamentation potentially prepares the fungus for future macrophage contacts since phagocytosis of hyphae is not only slower than internalization of yeast cells, but may lead to the formation of less microbicidal frustrated phagosomes [12], [126], [127]. Moreover, *C. albicans* even exploits immune molecules for its predictive adaptation: Immune mediators such as cytokines can induce, *via* the TOR nutrient sensing pathway, augmented adhesion, filamentation, and biofilm formation [271]. Additionally, *C. albicans* can sense niche-specific hormones, such as high estrogen levels in the vagina, to initiate virulence and immune evasion programs [272].

We believe that human pathogenic fungi possess even more predictive adaptation mechanisms to deal with immune surveillance mechanisms, and that this field deserves deeper investigation to achieve a better understanding of the fungal co-existence with the human host facilitated by immune evasion.

As the fungi and hosts are in a constant co-evolution, the host also "learns" to recognize fungal virulence attributes. For example, recent studies showed that the host can actively block potential detrimental hypha formation of colonizing *C. albicans* cells in the gut by producing IgA and IgG antibodies, which specifically target hyphae [7], [273], an activity that can be seen as an adaptive prediction of the host. Furthermore, the host can activate defensive responses, turning fungal virulence and immune evasion attributes into avirulence attributes [274]. Fungal pathogens can avoid that by attenuating immune recognition and damage induction, in order to stay “invisible”. This might be the case for candidalysin, the peptide toxin encoded by *ECE1* (see 3.3) [202], [206]. Among the *Candida* species, *C. albicans* shows the highest transcript levels of *ECE1* [275]. In contrast, *C. dubliniensis* and *C. tropicalis* do not strongly induce *ECE1* expression, preventing them from causing tissue damage [275]. This divergence between different species from the same genus suggests that there is an evolutionary pressure to reduce their visibility by producing less of a strongly immunogenic toxin, thus promoting immune evasion.

Interestingly, supporting this notion, a recent study demonstrated that strain-to-strain variations in *C. albicans ECE1* can affect immune recognition [276]. The authors found two different *ECE1* alleles conserved among *C. albicans* clinical strains, one associated with strong neutrophil attraction and immunopathology, whereas the other shows reduced host damage, cytokine release, and neutrophil recruitment [276]. Therefore, different *C. albicans* isolates might be heterogenic in their ability to evade the immune system. In line with this, diverse *C. albicans* isolates were found to differ in their capacity to elicit neutrophil recruitment and induce inflammation in the oral niche, indicating that the host orchestrates immune responses in a strain-specific manner [277].

Collectively, inter-species and inter-strain heterogeneity in the capacity to avoid immune activation underscores that there may be a strong evolutionary pressure to evolve different strategies and individual solutions, which help to escape the host immune system. As this could affect the outcome of fungal-host interactions, individual immune evasion strategies should be investigated further for the important fungal pathogens of humans.

## Funding

TL, LK, SB and BH receive funds by the Priority Program SPP 2225 “Exit strategies of intracellular pathogens”. BH is furthermore supported by the 10.13039/501100001659German Research Foundation (Deutsche Forschungsgemeinschaft - DFG) Cluster of Excellence “Balance of the Microverse”, the European Union Horizon 2020 grant agreement 847507 (HDM-FUN), and the Marie Sklodowska-Curie grant agreement 812969 (FunHoMic). MSG is supported by the DFG Emmy Noether Program (project no. 434385622 / GR 5617/1–1). BH and MG are furthermore supported by the DFG Collaborative Research Center (CRC)/Transregio (TRR) 124 FungiNet project C1.

## Data Availability

No data was used for the research described in the article.
